# Odor Discrimination by Similarity Measures of Abstract Odor Factor Maps from Electronic Noses

**DOI:** 10.3390/s18082658

**Published:** 2018-08-13

**Authors:** Weiqing Guo, Haohui Kong, Junzhang Wu, Feng Gan

**Affiliations:** 1 School of Chemistry, Sun Yat-Sen University, Guangzhou 510275, China; guowqing@mail2.sysu.edu.cn; 2 Technology Center, China Tobacco Guangdong Industrial Co., Ltd., Guangzhou 510385, China; konghh@gdzygy.com (H.K.); wujunzhang@gdzygy.com (J.W.)

**Keywords:** electronic nose, abstract odor factor maps, similarity measure, odor discrimination

## Abstract

The aim of this study is to improve the discrimination performance of electronic noses by introducing a new method for measuring the similarity of the signals obtained from the electronic nose. We constructed abstract odor factor maps (AOFMs) as the characteristic maps of odor samples by decomposition of three-way signal data array of an electronic nose. A similarity measure for two-way data was introduced to evaluate the similarities and differences of AOFMs from different samples. The method was assessed by three types of pipe and powder tobacco samples. Comparisons were made with other techniques based on PCA, SIMCA, PARAFAC and PARAFAC2. The results showed that our method had significant advantages in discriminating odor samples with similar flavors or with high VOCs release.

## 1. Introduction

In Nature, mammals discriminate odor through a complex process. First, the olfactory sensory neurons detected odor molecules, then axons on the neurons transmit the signal to olfactory bulb, and finally the olfactory bulbs process the signal to obtain the information of the odor [1]. Inspired by the odor discrimination process of mammals, researchers have developed artificial olfactory systems called electronic nose systems that contains perception, signal processing and recognition sections [2,3]. Although the discrimination ability of electronic nose systems is far beneath that of the mammalian olfactory system, these systems have been quickly applied to odor discrimination in many fields, such as agriculture [4,5,6], fishery [7,8,9], the food industry [10,11,12], disease diagnosis [13,14,15], environmental monitoring [16,17,18] and chemical safety [19,20] because of their objectivity, stability and durability. Compared with the traditional odor analysis techniques, such as gas chromatography and its hyphenated techniques [21,22], electronic nose systems show good application prospects because they are fast and sensitive, need simple pre-processing and operation steps, and can obtain overall information of the volatile components in samples.

However, commercial electronic nose systems usually have low selectivity [23] and cross-sensitivity [24], which limits their discrimination performance. Many data processing methods have been introduced to improve the discrimination performance of electronic noses [23,25,26,27,28,29,30,31,32]. The traditional data analysis methods for electronic noses are mainly based on bilinear models, such as PAC, SIMCA, DFA, KNN, SVM, etc. [25,26,27,28,29,30] Later, methods based on trilinear models were also applied to electronic nose data analysis, such as PARAFAC, PARAFAC2 and MOLMAP [23,31,32]. However, their performances were not quite satisfactory when electronic nose was applied to odor samples with high similarity or complexity. The reason could be that there were differences between the real response models of the sensor arrays and the mathematical models of these methods. Recently, researchers have attempted to solve the problem through new techniques, such as feature extraction (selection) [33,34,35,36], nonlinear modification [35,37], interference suppression algorithms [24] and so on. These techniques, to some extent, can improve the discrimination performance of electronic noses, but do not essentially solve the problem. New data processing methods are needed.

In this paper, we establish a new data processing method for the discrimination of similar or complex odor samples. A novel signal model based on the true response mechanism of a metal oxide semiconductor (MOS) sensor array had been developed in our previous work and had shown success in the discrimination of perfume samples [38]. Here, we develop a new algorithm for the decomposition of this signal mode, and further propose a conception of abstract odor factor maps (AOFMs). An AOFM is a two-way matrix that is reconstructed by the abstract factors decomposed from a three-way electronic nose signal data array. Thus, it can characterize the main odorous substances in the sample and be used as a characteristic map of an odor. A similarity measure method [39] for two-way data is introduced to evaluate the similarities and differences between the AOFMs of samples. The new method is applied to three types of pipe and powder tobacco samples, the results were compared with those of PCA, SIMCA, PARAFAC and PARAFAC2. The results showed that the new method outperformed the aforementioned methods, showing significant advantages in discriminating complex or similar odor samples.

## 2. Methodology

### 2.1. Algorithm for the Decomposition of Signal Model

We assume that an electronic nose system is made up of k sensors. When this system is used to measure n samples and the measuring time is t for each sample, a three-way data array ${\underline{R}}_{t\times k\times n}$ is obtained ${\underline{R}}_{t\times k\times n}.$ According to our previous study [38], each slice of ${\underline{R}}_{t\times k\times n}$ can be expressed as a two-way data $R_{t\times k,i}$, and be decomposed as follows:

(1) $$R_{t\times k,i}=C_{t\times k}N_{p\times \left(ck\right),i}\Gamma_{\left(ck\right)\times k}$$

Here, c is the number of abstract odor molecules and p is the number of factors. $C_{t\times p}$ is the concentration profiles of absorbed mass; $N_{p\times \left(ck\right),i}$ is a scaling matrix indicating the absorbed-amount ratios of sensors. $\Gamma_{\left(ck\right)\times k}$ is the matrix of odor characteristics which is band-diagonal matrix.

In our previous work, we temporarily decompose by a simple alternating least squares. Here we propose a new decomposition algorithm inspired by parallel factor analysis (PARAFAC).

(1) Step 1: obtain an initial $C_{t\times p}$. The slabs of ${\underline{R}}_{t\times k\times n}$ are stringed out horizontally as follows:
  (2) $$R_{\mathrm{col}}=\left[R_{t\times k,1}\;R_{t\times k,2}\;\ldots \;R_{t\times k,n}\right]$$
  Through principal component analysis of $R_{\mathrm{col}}$, the top p score vectors $t_{1},t_{2},\ldots ,t_{p}$ are used to construct $C_{t\times p}$:
  (3) $$C_{t\times p}=\left[t_{1}\;t_{2}\;\ldots \;t_{p}\right]$$
(2) Step 2: obtain an initial $\Gamma_{\left(ck\right)\times k}$. The slabs of ${\underline{R}}_{t\times k\times n}$ are stringed out vertically as follows:
  (4) $$R_{\mathrm{row}}=\left[\begin{array}{c} R_{t\times k,1}\\ R_{t\times k,2}\\ \vdots \\ R_{t\times k,n} \end{array}\right]$$
  The first c loadings $p_{1},p_{2},\ldots ,p_{c}$ that are obtained from principal component analysis of $R_{\mathrm{row}}$ are used to construct $\Gamma_{\left(ck\right)\times k}$ as follows:
  (5) $$\Gamma_{\left(ck\right)\times k}=\left[\begin{array}{cccc} \left(\begin{array}{c} p_{1}^{\left(1\right)}\\ p_{1}^{\left(2\right)}\\ \vdots \\ p_{1}^{\left(c\right)} \end{array}\right) & 0 & \ldots & 0\\ 0 & \left(\begin{array}{c} p_{2}^{\left(1\right)}\\ p_{2}^{\left(2\right)}\\ \vdots \\ p_{2}^{\left(c\right)} \end{array}\right) & \ldots & 0\\ \vdots & \vdots & \ddots & \vdots \\ 0 & 0 & \ldots & \left(\begin{array}{c} p_{k}^{\left(1\right)}\\ p_{k}^{\left(2\right)}\\ \vdots \\ p_{k}^{\left(c\right)} \end{array}\right) \end{array}\right]$$
(3) Step 3: calculate $N_{p\times \left(ck\right),i}$ by $C_{t\times p}$ and $\Gamma_{\left(ck\right)\times k}$:
  (6) $$N_{p\times \left(ck\right),i}={\left({\left(C_{t\times p}\right)}^{T}\left(C_{t\times p}\right)\right)}^{-1}{\left(\left(C_{t\times p}\right)\right)}^{T}R_{t\times k,i}{\left(\Gamma_{\left(ck\right)\times k}\right)}^{T}{\left(\left(\Gamma_{\left(ck\right)\times k}\right){\left(\Gamma_{\left(ck\right)\times k}\right)}^{T}\right)}^{-1}$$
(4) Step 4: calculate $C_{t\times p}$ by $N_{p\times \left(ck\right),i}$ and $\Gamma_{\left(ck\right)\times k}$:
  (7) $$N\Gamma =\left[N_{p\times \left(ck\right),1}\Gamma_{\left(ck\right)\times k}\;N_{p\times \left(ck\right),2}\Gamma_{\left(ck\right)\times k}\;\ldots \;N_{p\times \left(ck\right),n}\Gamma_{\left(ck\right)\times k}\right]$$
  (8) $$C_{t\times p}=R_{\mathrm{col}}{\left(N\Gamma \right)}^{T}{\left(N\Gamma {\left(N\Gamma \right)}^{T}\right)}^{-1}$$
(5) Step 5: calculate $\Gamma_{\left(ck\right)\times k}$ by $C_{t\times p}$ and $N_{p\times \left(ck\right),i}$:
  (9) $$CN=\left[\begin{array}{c} C_{t\times p}N_{p\times \left(ck\right),1}\\ C_{t\times p}N_{p\times \left(ck\right),2}\\ \vdots \\ C_{t\times p}N_{p\times \left(ck\right),n} \end{array}\right]$$
  (10) $$\Gamma_{\left(ck\right)\times k}={\left({\left(CN\right)}^{T}CN\right)}^{-1}{\left(CN\right)}^{T}R_{\mathrm{row}}$$
(6) Step 6: calculate residual sum of squares (SSR):
  (11) $$\mathrm{SSR}=\overset{n}{\underset{i=1}{\sum }}{\left(R_{t\times k,i}-C_{t\times p}N_{p\times \left(ck\right),n}\Gamma_{\left(ck\right)\times k}\right)}^{2}$$
(7) Repeat Steps (3), (4), (5) and (6) until SSR reaches preset value.

In the decomposition process, three constraints are implemented (1) nonnegative constraints are implemented to matrices C, N and Γ; (2) normalization in unit length and unimodal constraint are implemented to each column of matrix C; (3) a band-diagonal constraint is implemented to matrix Γ.

### 2.2. The Construction of AOFMs

When the response matrix $R_{t\times p,i}$ of an odor sample is decomposed into p factors according to the Equation (1), its p odor maps can be re-constructed in following way:

(12) $$R_{j}^{*}=c_{j}n_{j}^{T}\Gamma_{\left(ck\right)\times k},\;j=1,2,\ldots ,p$$

where, $R_{j}^{*}$ is the jth odor map, $c_{j}$ is the jth column of matrix $C_{t\times p}$, $n_{j}^{T}$ is the transpose of the jth row of matrix $N_{p\times \left(ck\right),i}$. A two-way matrix $R^{*}$, the AOFMs, is then constructed as follows:

(13) $$\mathrm{AOFM}\;\sim \;R^{*}=\left[\begin{array}{c} R_{1}^{*}\\ R_{2}^{*}\\ \vdots \\ R_{p}^{*} \end{array}\right]$$

### 2.3. Similarity Measure of AOFMs

One of the authors of this paper had put forward a similarity measure method for two-way data [39]. A brief explanation of the method is given here. If the AOFMs of two odor samples have the forms of $R_{A}^{*}$ and $R_{B}^{*}$, their differences can be calculated as follows:

(14) $$\Delta R^{*}=R_{A}^{*}-R_{B}^{*}$$

A statistic is constructed as follows, which is the mean value of $\Delta R^{*}$:

(15) $$T=\frac{1}{m\times n}\overset{n}{\underset{j=1}{\sum }}\overset{m}{\underset{i=1}{\sum }}\Delta R^{*}\left(i,j\right)$$

where, m and n are the row and column of $\Delta R^{*}$. When T is known, a hypothesis test and its posterior probability are expressed as follows:

(16) $$\{\begin{array}{ll} H_{0} & \Delta R^{*}=R_{A}^{*}-R_{B}^{*}=0\\ H_{1} & \Delta R^{*}=R_{A}^{*}-R_{B}^{*}\neq 0 \end{array}$$

(17) $$POR=\frac{p(H_{0}|T)}{p(H_{1}|T)}=\left[\frac{p\left(H_{0}\right)}{p\left(H_{1}\right)}\right]\left[\frac{p(T|H_{0})}{p(T|H_{1})}\right]$$

Here, *POR* is the posterior odds ratio. Equation (17) can be converted to POR=αLR. The mathematical expressions of α and LR are Equations (S1) and (S2) (supporting information), respectively. Parameter LR is the likelihood ratio of two samples and α represents prior probabilities. LR can be calculated by Equation (S4) and α can be calculated by the AOFMs of samples of a training set, which is described in reference [39] or supporting information. To discriminate an odor sample in prediction set, its AOFMs should be compared with the mean AOFMs in the training set. If POR<1, the two odor samples are statistically different; If POR>1, they are considered with same odor. In this way, odor samples can be discriminated.

For the prediction of an unknown samples, we should firstly calculate the T between the AOFMs of unknown samples and the mean AOFMs of training set samples according to Equations (14) and (15). When T is known, the parameter LR can be calculated by Equation (S4). Since the parameter α of the training set samples is known, the values of *POR* between the unknown samples and training set samples are calculated according to POR=αLR. Finally, the similarities between the samples are judged by the value of *POR*.

## 3. Materials and Methods

### 3.1. Instruments

All the data were obtained from an αFox-4000 electronic nose equipped with Alpha Multi Organoleptic System (Alpha MOS, Toulouse, France). The system contains 18 MOS sensors that are placed in three chambers, a HS-100 auto-sampler, and an AG2301 high pure air generator.

### 3.2. Sample and Measurement Condition

Three types of pipe tobacco and tobacco powder samples with similar flavor were provided by the Technology Center of Tobacco Guangdong Industrial Co., Ltd. (Guangzhou, China). The pipe tobacco samples are labeled as Pipe Tobacco I, Pipe Tobacco II and Pipe Tobacco III, while powder samples are labeled Tobacco Smalls I, Tobacco Smalls II and Tobacco Smalls III. Each group of the samples included 18 samples, and hence in total there were 54 Pipe Tobacco and 54 Tobacco Smalls.

The measurement conditions were optimized by experiments. A certain amount of pipe/powder was taken into a 10 mL glass vial and then sealed as a sample. The sample was placed in HS-100 auto-sampler and equilibrated for 600 s. The equilibrating temperature for Pipe Tobacco was 60 °C, and for Tobacco Smalls was 35 °C. After that, 2500 μL of the headspace gas in the vial was injected into the syringe by a sampling pump at the speed of 350 mL/min, and then injected into the electronic nose system along with high pure air which acted as carrier gas at the speed of 500 mL/min. The response of system in first 100 s was used for data analysis. It was recorded every 1 s. The sensors were cleaned up by high pure air for 1080 s after each measurement.

### 3.3. Data Processing

Pre-processing of data was made before the signal model was applied to the data. The pre-processing methods are shown in Equations (S5) and (S6) (Supporting Information). After the measurement of each sample, a two-way data with the size of 100 × 18 (time × sensor) was obtained. Thus, 18 samples provided 18 data arrays for each group of samples. Finally, the 18 two-way data arrays were arranged into a three-way data with the size of 100 × 18 × 18 (time × sensor × sample).

To obtain the optimal result of the model, the parameters *c* and *p* should be optimized. The optimized range of parameter *c* was from 1 to 3, and *p* was from 1 to 5. The data were decomposed based on the signal model by setting different *c* and *p*. The sum of squares of residual (SSR) was the optimization criterion. The data analysis for PCA and SIMCA were performed using the AlphaSoft, Version 12.42 (Alpha MOS). The data analysis for other methods were implemented using Octave, Vision 4.0.3.

## 4. Results and Discussion

### 4.1. The AOFMs of Tobacco Smalls and Pipe Tobacco

**Figure 1:**
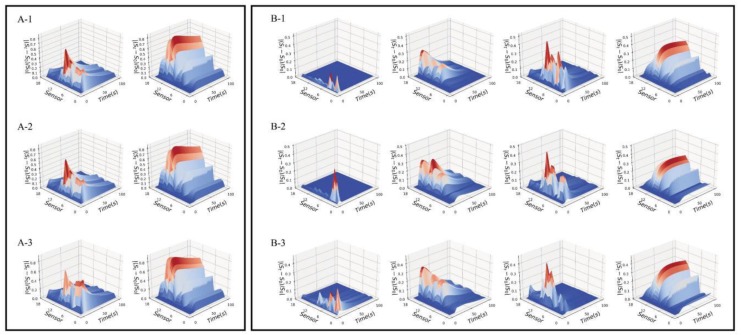
The odor maps of three types of Pipe Tobacco (**A**) and Tobacco Smalls (**B**). (**A**-**1**) Pipe Tobacco I; (**A**-**2**) Pipe Tobacco II; (**A**-**3**) Pipe Tobacco III; (**B**-**1**) Tobacco Smalls I; (**B**-**2**) Tobacco Smalls II; (**B**-**3**) Tobacco Smalls III.

The pre-processing method and proposed decomposition algorithm had been applied to the signal data of Pipe Tobacco and Tobacco Smalls samples. The decomposition results with different parameters *c* and *p* are shown in Tables S1 and S2. It showed that the optimum value of *c* for all tobacco samples was 2. Meanwhile, the optimal *p* value of Pipe Tobacco samples was 2, while the optimal *p* value of Tobacco Smalls samples was 4. The data of Pipe Tobacco and Tobacco Smalls samples were decomposed with the optimal value of parameters, and we obtained their odor maps which were shown in Figure 1.

**Figure 2:**
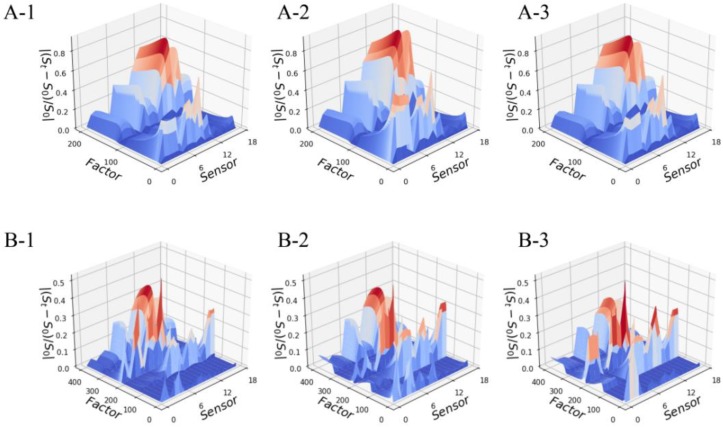
The two-way AOFM of three Pipe Tobacco samples (**A**) and three Tobacco Smalls samples (**B**): (**A**-**1**) Pipe Tobacco I; (**A**-**2**) Pipe Tobacco II; (**A**-**3**) Pipe Tobacco III; (**B**-**1**) Tobacco Smalls I; (**B**-**2**) Tobacco Smalls II; (**B**-**3**) Tobacco Smalls III.

**Figure 3:**
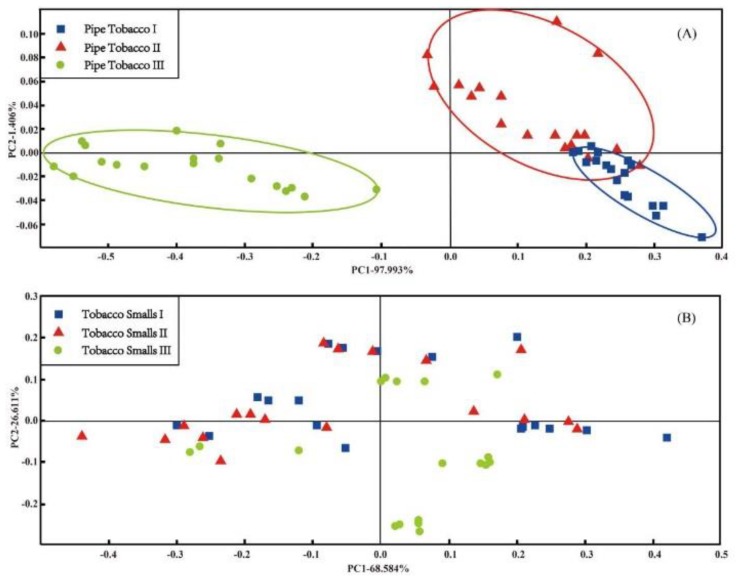
The PCA score plots of three types of Pipe Tobacco samples (**A**) and Tobacco Smalls (**B**).

It should be explained that the number of odor maps depends on the value of model parameter *p* (see Equation (12)). As the *p* value of Pipe Tobacco samples was 2, each Pipe Tobacco sample had obtained two odor maps which are shown in Figure 1(A-1–A-3). For Tobacco Smalls samples, the value of *p* was 4 and thus all of them gave four odor maps which are shown in Figure 1(B-1–B-3). We noted that odor maps of the Tobacco Smalls samples were more than Pipe Tobacco. This result can be explained. Tobacco Smalls were powder samples and thus they had larger specific surface area than Pipe Tobacco samples which were filamentous. Larger specific surface area was more conducive to the release of volatile organic compounds (VOCs). Therefore, the odors of Tobacco Smalls samples were more complex than Pipe Tobacco and need more odor maps to represent their feature information.

We could distinguish the different odor samples by visually comparing their odor maps one by one. However, such a strategy was relatively time-consuming and lacked any objective criteria. Here, we reconstruct the odor maps into a two-way AOFM to comprehensively express the odor characteristics of each sample. The AOFMs of all Pipe Tobacco and Tobacco Smalls samples are shown in Figure 2, and we would use a similarity measure to evaluate their differences instead of visual observation.

### 4.2. Similarity Measure of AOFM

In order to evaluate the differences between the odors of the Pipe Tobacco samples, we set the AOFMs of each group of tobacco samples as the training set, and AOFMs of the other two groups as the prediction set. The similarities of AOFMs between prediction and training set samples were calculated (represented by the values of *POR*) as the criteria for the discrimination of their odor. The similarity measure results of AOFMs of Pipe Tobacco and Tobacco Smalls are summarized in Table 1.

When the AOFMs of Pipe Tobacco I were set as the training set, we got *POR* < 1 for both Pipe Tobacco II and III. This meant that Pipe Tobacco II and III were different from I, and thus Pipe Tobacco I samples can be distinguished from them. Pipe Tobacco II and III also can be discriminated by the same method. We noted when the AOFMs of Pipe Tobacco I were used as the training set, the *POR* values of AOFMs of Pipe Tobacco III were less than those of Pipe Tobacco II. Similarly, when the AOFMs of Pipe Tobacco II were used as the training set, the *POR* values of AOFMs of Pipe Tobacco III were also much less than those of Pipe Tobacco I. This finding supported the notion that the AOFMs of Pipe Tobacco I and II were similar to those of Pipe Tobacco III. Actually, Pipe Tobacco I, II and III were tobaccos with strong flavor. Pipe Tobacco I and II were from the same series while Pipe Tobacco III was from a different series. Hence, the flavors of Pipe Tobacco I and II were more similar than III and the similarity measure result was consistent with this fact.

The same method was also applied to calculate the *POR* values of the AOFMs of the Tobacco Smalls samples. We found that the *POR* values obtained from the six settings were also less than 1 and infinitely close to 0.00. This indicated that there were great differences between the prediction and the training samples. The results indicated that these three groups of Tobacco Smalls samples can be identified correctly from each other. Cellini et al. [40] noted that the discrimination performance of electronic nose might be weakened when it was applied to samples with high VOCs release. In this case, our method can effectively solve this problem.

### 4.3. Other Methods

In order to assess the advantages and disadvantages of our new method, the result is compared with those from PCA, SIMCA, PARAFAC and PARAFAC2.

#### 4.3.1. PCA

PCA is a pattern recognition method contained in the αFox4000 software. It had been used for analyzing measured data of tobacco samples, and the result is shown in Figure 3. 

Figure 3A is the PCA score plot of the Pipe Tobacco samples. It shows that there were certain differences among the odors of the three groups Pipe Tobacco samples. The points of Pipe Tobacco I and Pipe Tobacco II were obviously overlapped with each other, but Pipe Tobacco III points were separated from the other two Pipe Tobacco groups. This result implied that PCA can effectively discriminate Pipe Tobacco III, but cannot discriminate Pipe Tobacco I and II as they were more similar. For the Tobacco Smalls samples, the result is quite unsatisfactory. The three groups of Tobacco Smalls points were overlapped completely, and none of them can distinguished from each other (Figure 3B). The high VOCs release prevented an effective electronic nose discrimination in these results. According to Cellini et al. [40], too many VOCs released from Tobacco Smalls sample may be prone to producing a high background noise. This situation finally resulted that the samples of three groups Tobacco Smalls cannot be distinguished in the PCA score plot.

#### 4.3.2. SIMCA

**Figure 4:**
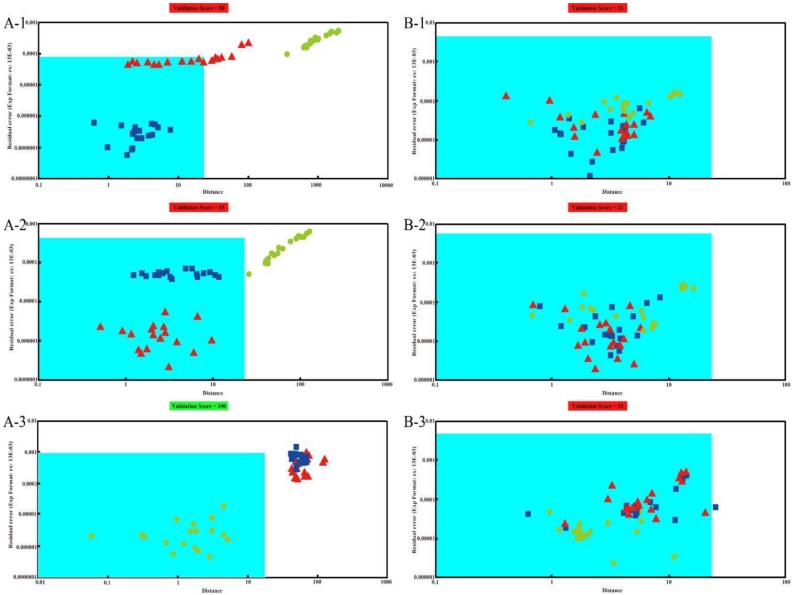
The SIMCA results of three types of Pipe Tobacco samples (**A**) and Tobacco Smalls (**B**). The training sets are Pipe Tobacco I (**A**-**1**), Pipe Tobacco II (**A**-**2**), Pipe Tobacco III (**A**-**3**), Tobacco Smalls I (**B**-**1**), Tobacco Smalls II (**B**-**2**), and Tobacco Smalls III (**B**-**3**), respectively.

The SIMCA method is another data analysis method contained in αFox4000 software which is used for cluster analysis. Figure 4(A-1–A-3) are the SIMCA results of Pipe Tobacco while Figure 4(B-1–B-3) are the results of the Tobacco Smalls. The area with blue background in Figure 4 is the identification area. When prediction samples fall in the identification area, they would be considered the same as the training samples, otherwise they would be considered as different odors. In Figure 4(A-1), the samples of Pipe Tobacco I were used as training set, and the samples of Pipe Tobacco II and III were used as the predicted set. We can find that some of Pipe Tobacco II samples fell in the identification area, and were wrongly recognized as Pipe Tobacco I. Meanwhile, all samples of Pipe Tobacco III fell away from the identification area, and hence were correctly discriminated from Pipe Tobacco I. The training samples of Figure 4(A-2) were Pipe Tobacco II while prediction samples were Pipe Tobacco I and III. The result showed that all Pipe Tobacco I samples were wrongly recognized as Pipe Tobacco II, but the Pipe Tobacco III samples were fell outside the identification area and were correctly distinguished from Pipe Tobacco II. When Pipe Tobacco III samples were used as training set (Figure 4(A-3)), both the Pipe Tobacco I and II samples stayed away from the identification area and were correctly distinguished. Therefore, we can find the SIMCA results were similar to the PCA ones, and inferior to the similarity measure of AOFMs. The Pipe Tobacco III samples can be distinguished from the other two groups of Pipe Tobacco, but Pipe Tobacco I and II samples cannot be distinguished from each other by the SIMCA method due to their higher similarity. The training samples of Figure 4(B-1–B-3) were Tobacco Smalls I, II and III, respectively. From these three subfigures, we can see that almost all the samples had fallen into the recognition area and were considered as the same samples. Particularly in Figure 4(B-1,B-2), all points of samples were disordered. The points representing the same group of Tobacco Smalls were diffuse, and those representing different groups were mixed. In Figure 4(B-3), though all samples also were in the recognition area and misjudged as the same samples, samples of Tobacco Smalls III were still clustered and away from those of Tobacco Smalls I and II. Thus, even though SIMCA also cannot effectively discriminate between these three groups of Tobacco Smalls, its results were better than PCA. The reason may be that SIMCA is more effective in extracting feature information as it is a supervised learning method and PCA is an unsupervised one.

#### 4.3.3. PARAFAC and PARAFAC2

**Figure 5:**
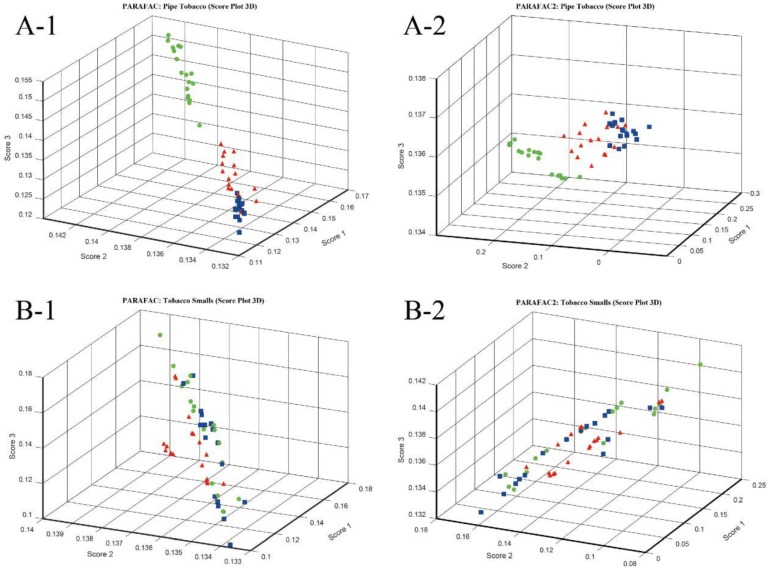
3D score plot with top three components of Pipe Tobacco and Tobacco Smalls by PARAFAC and PARAFAC2. (**A**-**1**) Pipe Tobacco, PARAFAC; (**A**-**2**) Pipe Tobacco, PARAFAC2; (**B**-**1**) Tobacco Smalls, PARAFAC; (**B**-**2**) Tobacco Smalls, PARAFAC2.

Considering that the data of electronic nose can be expressed as three-way data array, three-way resolution methods, such as parallel factor analysis (PARAFAC), are also applicable options for analyzing electronic nose data. In this paper, PARAFAC and PARAFAC2 were used to discriminate Pipe Tobacco and Tobacco Smalls samples, and their number of components were optimized. Figure S1 shows the trends of SSR versus the number of components when PARAFAC and PARAFAC2 were implemented for both the Pipe Tobacco and Tobacco Smalls samples. The results show that three principal components were appropriate for these PARAFAC and PARAFAC2 models. Figure 5(A-1,A-2) are the 3D score plots of PARAFAC and PARAFAC2 for Pipe Tobacco samples. The figures showed that Pipe Tobacco III samples were clustered but were separated from the Pipe Tobacco I and II ones. However, for the samples of Pipe Tobacco I and II, some of the points were overlapped. These results indicated that both PARAFAC and PARAFAC2 could distinguish Pipe Tobacco III but cannot distinguish Pipe Tobacco I and II which were more similar. The results of PARAFAC and PARAFAC2 for Tobacco Smalls were even worse. Their 3D score plots are shown in Figure 5(B-1,B-2). The samples of the same groups of Tobacco Smalls were diffused and those of different groups were mixed in these two subfigures. Thus, PARAFAC and PARAFAC2 also had difficulties to distinguish Tobacco Smalls samples as they were more complex.

### 4.4. Comparison

Table 2 lists the discrimination performance of the five methods. We can find that similarity measure of AOFMs outperformed PCA, SIMCA, PARAFAC and PARAFAC2. Comparing with the results of the aforementioned methods, the similarity measure of AOFMs can improve the discrimination performance of electronic noses in their applications to samples with similar flavors (Pipe Tobacco I and II) or with high VOCs releases (Tobacco Smalls samples).

## 5. Conclusions

An effective method for measuring the similarity of complex odor samples has been established in this paper. The success is attributed to the introduction of the AOFMs that can represent all the information of an odor sample and show more details of an odor sample than its original signal form. The AOFMs also provide the basis to apply a two-way similarity measurement technique, which makes the odor discrimination more objective and effective than other techniques such as PCA, SIMCA, and PARAFAC when complicated odor samples are encountered.

## Figures and Tables

**Table 1:** The similarity measure results of AOFMs of Pipe Tobacco and Tobacco Smalls.

No.	Training Set	Predicted Set	The Value of *POR*	Whether Be Separated
Pipe Tobacco	I	II	2.87 × 10^−209^~9.14 × 10^−88^	Yes
		III	→0.00 ^1^	Yes
	II	I	5.32 × 10^−59^~1.81 × 10^−14^	Yes
		III	→0.00 ^1^	Yes
	III	I	→0.00 ^1^	Yes
		II	→0.00 ^1^	Yes
Tobacco Smalls	I	II	→0.00 ^1^	Yes
		III	→0.00 ^1^	Yes
	II	I	→0.00 ^1^	Yes
		III	→0.00 ^1^	Yes
	III	I	→0.00 ^1^	Yes
		II	→0.00 ^1^	Yes

^1^ →0.00 means the value of *POR* is infinitely close to 0.00.

**Table 2:** The discrimination performance of the five methods.

Label		Separation of Samples ^1^				
		Similarity Measure of AOFM	PCA	SIMCA	PARAFAC	PARAFAC2
Pipe Tobacco	I-II	+	−(+)	−	−(+)	−(+)
	I-III	++	++	++	++	++
	II-III	++	++	++	++	++
Tobacco Smalls	I-II	++	−	−	−	−
	I-III	++	−	−	−	−
	II-III	++	−	−	−	−

^1^ Separation of different tobacco samples evaluated from results of five methods. −: No separation; −(+): samples are partially separated; +:samples are separated; ++: samples are well separated.

## Supplementary Materials

The following are available online at http://www.mdpi.com/1424-8220/18/8/2658/s1, Equations (S1)–(S6), Table S1: The SSR of the Pipe Tobacco samples, Table S2: The SSR of the Tobacco Smalls samples, Figure S1: SSR of Pipe Tobacco and Tobacco Smalls samples along with growth of components by PARAFAC and PARAFAC2.
