# Supplementary material for: Odor Discrimination by Similarity Measures of Abstract Odor Factor Maps from Electronic Noses
Source: Sensors (Basel). 2018 Aug 13;18(8):2658. doi: 10.3390/s18082658 (PMC6111723; doi:10.3390/s18082658)
Supplement: Supplementary file 1 [file sensors-18-02658-s001.pdf]

Supporting Information

Odor Discrimination by Similarity Measure of Abstract Odor Factor Maps From Electronic Nose

—Supporting Information—

Weiqing GUO [1], Haohui KONG [2] , Junzhang WU [2] and Feng GAN [1,*]

[1] School of Chemistry, Sun Yat-Sen University, Guangzhou 510275, PR China; guowqing@mail2.sysu.edu.cn (WG); cesgf@mail.sysu.edu.cn(FG);
[2] Technology Center, China Tobacco Guangdong Industrial Co., Ltd., Guangzhou 510385, PR China; konghh@gdzygy.com(HK);wujunzhang@gdzygy.com(JW);
* Correspondence: cesgf@mail.sysu.edu.cn;

Abstract

There were several information provided in the Supporting Information including mathematical expression for similarity measure of AOFMs, preprocessing of the data, optimization of parameter *c* and *p* in signal model and optimization of components number for PARAFAC and PARAFAC2.

Similarity measure of AOFMs

$$\alpha = \frac{p(\mathrm{H}_0)}{p(\mathrm{H}_1)} \tag{S1}$$

$$LR = \frac{p(T \mid \mathrm{H}_0)}{p(T \mid \mathrm{H}_1)} \tag{S2}$$

$$POR = \frac{p(\mathrm{H}_0 \mid T)}{p(\mathrm{H}_1 \mid T)} \tag{S3}$$

Parameters *LR* can be calculated by the AOFMs of samples according to Eq.(S4).

$$LR = \frac{p(T \mid \mathrm{H}_0)}{p(T \mid \mathrm{H}_1)} = \exp\left[-\left(\frac{mn}{4}\right)\left(6\sqrt{\frac{2}{mn}}T - \frac{18}{mn}\right)\right] \tag{S4}$$

The parameters α can be calculated by the AOFMs of samples in training set. As all the samples in training set are considered as statistically undifferentiated, their AOFMs should all satisfy $POR \geq 1$ when they are compared with their mean abstract odor factor maps. That means the value of α is equal to maximum when the training set data satisfy $\alpha LR \geq 1$.

Preprocessing of the data

The pre-processing method provided by α-Fox-4000 electronic nose system was as follow:

$$r = \frac{S_t - S_0}{S_0} \tag{S5}$$

where S_t is the conductance on a sensor at time t; S_0 is the initial conductance on the sensor. To obtain positive signals, a further processing was done as follows:

$$r = \left| \frac{S_t - S_0}{S_0} \right| \tag{S6}$$

Optimization of *c* and *p*

Pipe Tobacco samples

The SSR of three types of Pipe Tobacco samples are showed in Table S1, in which c is set from 1 to 3 and p is from 1 to 5.

Table S1 The SSR of the Pipe Tobacco samples

		p=1	p=2	p=3	p=4	p=5
Pipe Tobacco I	c=1	96.7629	6.3792	14.9712	12.0148	12.7580
	c=2	96.7629	4.8290	14.0438	14.9014	11.9821
	c=3	96.7642	4.4430	12.1753	13.0531	13.2112
Pipe Tobacco II	c=1	208.9450	19.0570	23.9970	25.3260	26.0760
	c=2	208.9450	10.3710	26.7820	24.1630	22.8390
	c=3	208.9450	14.7720	19.9750	23.3980	20.3580

Pipe Tobacco III	c=1	127.7300	18.5122	22.6276	20.7371	19.6808
	c=2	127.7302	7.6100	20.2553	19.5995	16.1550
	c=3	127.7301	7.1854	8.8253	18.3942	16.5193

The SSR of three types of Pipe Tobacco were basically the same when *c* was set as 2 or 3, and were less than those when *c* was set as 1. To avoid over-fitting, the most optimal value of c is 2. When *p* was set as 2, the residual errors were the least. Therefore, the most optimal value of *p* was 2 .

Tobacco Smalls samples

The SSR of three types of Tobacco Smalls samples are shown in Table S2, in which *c* is set from 1 to 3 and *p* is from 1 to 5.

Table S2 The SSR of the Tobacco Smalls samples

		p=1	p=2	p=3	p=4	p=5
Tobacco Smalls I	c=1	155.54	34.03	25.29	13.00	12.06
	c=2	155.54	28.25	22.70	10.70	9.14
	c=3	155.54	29.77	22.40	13.17	13.91
Tobacco Smalls II	c=1	165.37	29.95	23.21	18.36	15.17
	c=2	165.37	24.50	19.80	13.98	14.57
	c=3	165.37	24.90	20.23	15.14	15.91

Tobacco Smalls III	c=1	187.34	26.00	21.65	12.71	14.63
	c=2	187.34	24.59	19.95	8.64	10.18
	c=3	187.34	25.17	20.49	13.07	13.58

According to the Table S2, the SSR of the three types Tobacco Smalls were basically the same when *c* was set as 2 or as 3, and were less than those when *c* was set as 1. To avoid over-fitting, the most optimal value of *c* is 2. When t*p* was set as 4 and 5, there is not significant difference between their SSR. Thus, the most optimal value of *p* was 4.

Number of components for PARAFAC and PARAFAC2

Fig.S1 showed SSR of Pipe Tobacco and Tobacco Smalls samples along with growth of components. According to the trend, SSR had reach small enough when the number of components was set as 3. When the number of components increased from 3 to 5, SSR did not significantly decrease. Avoiding over-fitting, the optimal value was 3.

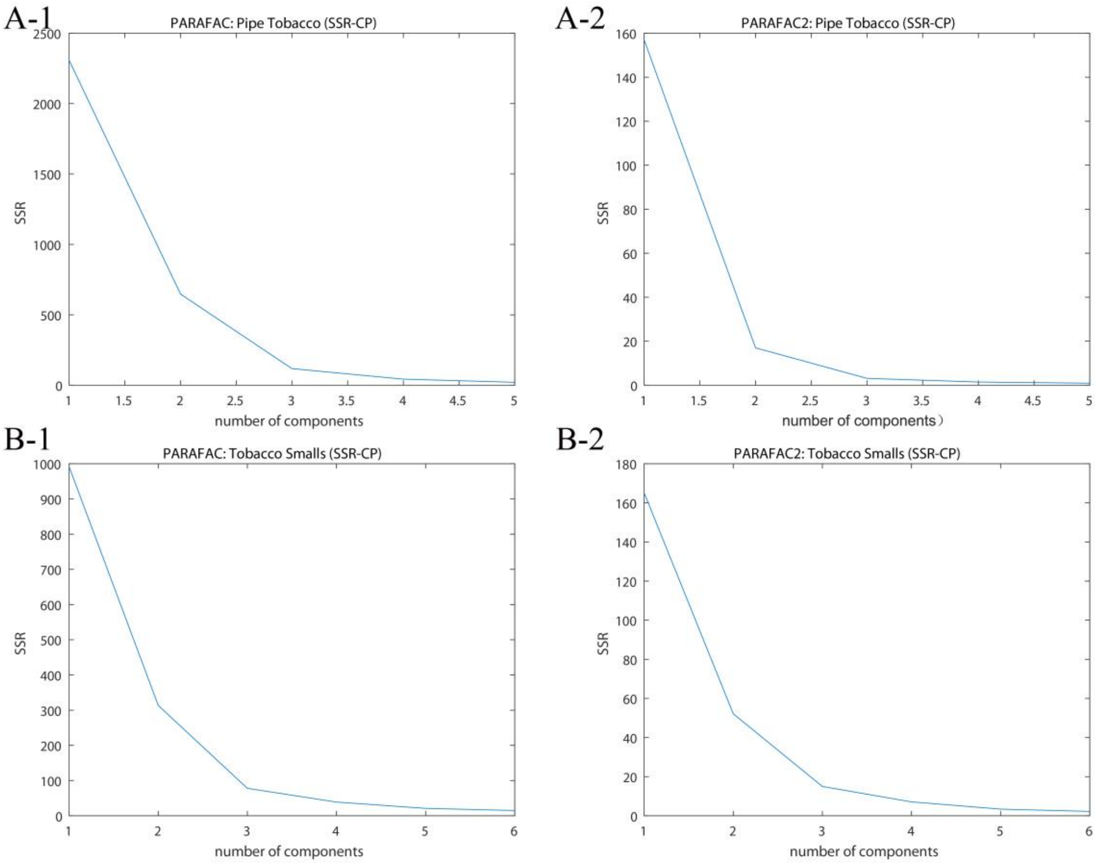

Figure S1. SSR of Pipe Tobacco and Tobacco Smalls samples along with growth of components by PARAFAC and PARAFAC2. (A-1) Pipe Tobacco, PARAFAC; (A-2) Pipe Tobacco, PARAFAC2; (B-1) Tobacco Smalls, PARAFAC; (B-2) Tobacco Smalls, PARAFAC2.
